# Determination of a Tentative Epidemiological Cut-Off Value (ECOFF) for Dalbavancin and *Enterococcus faecium*

**DOI:** 10.3390/antibiotics10080915

**Published:** 2021-07-27

**Authors:** Robert E. Weber, Carola Fleige, Franziska Layer, Bernd Neumann, Michael Kresken, Guido Werner

**Affiliations:** 1 National Reference Centre (NRC) for Staphylococci and Enterococci, Division of Nosocomial Pathogens and Antibiotic Resistances, Department of Infectious Diseases, Robert Koch Institute, Wernigerode Branch, 38855 Wernigerode, Germany; weberr@rki.de (R.E.W.); fleigec@rki.de (C.F.); layerf@rki.de (F.L.); neumannb@rki.de (B.N.); 2 Antiinfectives Intelligence, 51105 Cologne, Germany; michael.kresken@antiinfectives-intelligence.de; 3 University of Applied Sciences, 50968 Cologne, Germany

**Keywords:** dalbavancin, *E. faecium*, antibiotic resistance, ECOFF, MIC determination

## Abstract

Dalbavancin is a lipoglycopeptide antibiotic that shows potent activity against Gram-positive bacteria. It circumvents *vanB*-type glycopeptide resistance mechanisms; however, data on the in vitro activity of dalbavancin for *Enterococcus faecium* (*E. faecium*) are scarce, and thus, no breakpoints are provided. In recent years, there has been a continuing shift from *vanA*-type to *vanB*-type vancomycin-resistance in enterococci in Central Europe. Therefore, we aimed to investigate the in vitro activity of dalbavancin against different *van*-genotypes, with particular focus on *vanB*-type *E. faecium*. Dalbavancin susceptibility was determined for 25 *van*-negative, 50 *vanA*-positive, and 101 *vanB*-positive clinical *E. faecium* isolates (typed by cgMLST). Epidemiological Cut-Off Values (ECOFFs) were determined using ECOFFinder. For *vanB*-type *E. faecium* isolates, dalbavancin MICs were similar to those of vancomycin-susceptible isolates reaching values no higher than 0.125 mg/L. ECOFFs for *van*-negative and *vanB*-positive isolates were 0.5 mg/l and 0.25 mg/L respectively. In contrast, *E. faecium* possessing *vanA* predominantly showed dalbavancin MICs >8 mg/L, therefore preventing the determination of an ECOFF. We demonstrated the potent in vitro activity of dalbavancin against vancomycin-susceptible and *vanB*-type *E. faecium*. On the basis of the observed wildtype distribution, a dalbavancin MIC of 0.25 mg/L can be suggested as a tentative ECOFF for *E. faecium*.

## 1. Introduction

Enterococci are intestinal commensals in many animals and humans. However, few members of this genus are also important nosocomial pathogens that are capable of causing severe infections in critically ill and immunocompromised patients. In particular, *Enterococcus faecium* isolates have received increased public health attention due to a dramatic increase of related infections and the accumulation of antibiotic resistance determinants [1]. The European Antimicrobial Resistance Surveillance Network (EARS-Net) has shown an overall increasing trend in the proportion of vancomycin resistant enterococci (VRE = *E. faecium*) over the last few years [2,3]. However, no distinct geographical patterns could be observed across Europe, and even in countries with similar VRE rates, trends may differ with regard to the dissemination of different strain backgrounds and *van* genotype dynamics. Countries such as Denmark and Switzerland showed rising VRE trends, mainly due to an increased incidence of *vanA*-type *E. faecium* clonal types like ST203 and ST796 [4,5,6]. In contrast, other countries such as Germany and Poland reported a high prevalence of *vanB*-type resistance, which was preferably found among the *E. faecium* isolates of ST78 or ST117 [7,8,9].

Most VRE remain susceptible against last resort antibiotics such as linezolid, daptomycin (high dose), and tigecycline, but their use for patient treatment is constrained by several factors [10]. In addition to pharmacological and regulatory limitations, resistances to these novel antibacterials are increasingly reported [11,12,13,14,15]. In the case of *vanB*-type VRE, teicoplanin may be considered an effective treatment alternative. However, at least in Germany, the substance is not widely used, and published experience reports warn against the development of a resistance to teicoplanin under therapy [16].

For lipoglycopeptides such as telavancin, dalbavancin, and oritavancin, different in vitro activities against VRE have been published [17,18]. Oritavancin is the only lipoglycopeptide that is active against all VRE, regardless of the *van*-genotype that is present [19]. The antibiotic has been approved by the FDA and EMA for the treatment of acute bacterial skin and skin-structure infections (ABSSSI) but is only available on request or as an import in Europe. Telavancin and dalbavancin seem to be only effective against *vanB*-type VRE. Additionally, telavancin has been approved for nosocomial and ventilator-associated pneumonias and as such, is not of interest as an VRE agent.

Dalbavancin (Xydalba^®^) is approved in several European countries, including Germany, for treating ABSSSI and has been included in a clinical trial to demonstrate its efficacy in the treatment of invasive infections such as infective endocarditis and complicated bacteremia. However, the trial was terminated, and the results have not yet been published (trial identifier: NCT03148756; study ID: DAL-MD-09).

With the present study, we investigated the in vitro activity of dalbavancin, especially against *vanB*-type VRE. Furthermore, we aimed to determine a tentative ECOFF for *E. faecium* isolates, since EUCAST provides neither an ECOFF for *E. faecium* nor distinguishes between vancomycin-susceptible enterococci (VSE) and VRE. Our re-assessment also considered that most of the available in vitro studies on dalbavancin efficacy are now more than 10 years old and contained only a few VRE strains, all of which were poorly characterized with respect to species identification and *van*-genotyping [20,21].

For the present study, we compiled a large, comprehensive collection of invasive clinical *E. faecium* isolates that contained both vancomycin-susceptible and vancomycin-resistant isolates of the *vanA*- and *vanB*-types. All isolates originated from submissions to the German National Reference Centre (NRC) for Enterococci between January 2018 and December 2019. The collection was geographically diverse and represented the most common clonal types and *van* genotypes in Germany. Dalbavancin MICs were determined using broth microdilution (BMD) and a commercial gradient strip test. Next Generation Sequencing (NGS) data were available for all of the strains included in this study.

## 2. Results

### 2.1. Molecular Typing of the Strain Collection

The strain collection contained a total of 101 *vanB*-positive, 50 *vanA*-positive, and 25 *van*-negative (vancomycin-susceptible) *E. faecium* isolates belonging to 16 MLST and 74 cgMLST types (Supplementary Table S1 and Figure S1). The most frequent MLST types were ST117 (*n* = 89; 50.6%) and ST80 (*n* = 36; 20.5%); the most frequent cgMLST types were CT71 (ST117) (*n* = 39; 22.2%) and CT894 (ST80) (*n* = 8; 4.5%) (Supplementary Figure S1).

### 2.2. Determination of Dalbavancin MICs by BMD Testing

**Figure 1:**
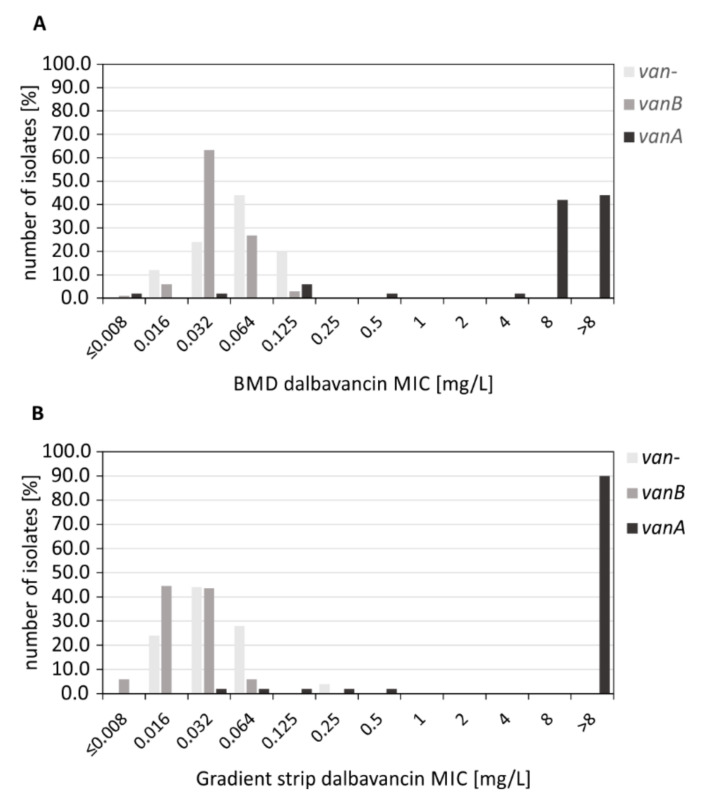
Distribution of dalbavancin MICs. MICs were obtained for 25 vancomycin-susceptible, 50 *vanA*-positive, and 101 *vanB*-positive *E. faecium* isolates using BMD (**A**) and MIC test strips (**B**). The number of isolates with corresponding MICs is given in %. Gradient strip MICs were both extrapolated to the next double dilution value equivalent to values for BMD and downsized to a maximum of >8 mg/L. The MIC breakpoint for dalbavancin for *E. faecalis* according to CLSI is ≤0.25 mg/L.

Vancomycin-susceptible and *van*-negative *E. faecium* isolates (*n* = 25) demonstrated dalbavancin MIC_50_ and MIC_90_ values of 0.064 mg/L and 0.125 mg/L, respectively (Table 1). The ECOFF was 0.5 mg/L (Table 1, Figure 1A). Dalbavancin MICs of *vanB*-positive *E. faecium* isolates (*n* = 101) tended to be one dilution below from what could be determined for *van*-negative *E. faecium* isolates (MIC_50_ = 0.032 mg/L, MIC_90_ = 0.064 mg/L) (Table 1). The ECOFF for the *vanB* isolates was 0.25 mg/L (Table 1, Figure 1A). According to the CLSI breakpoint for *E. faecalis* (S ≤ 0.25), all *van*-negative and *vanB*-positive *E. faecium* isolates would be categorized as dalbavancin-susceptible. *E. faecium* possessing *vanA* (*n* = 50) demonstrating a MIC_50_ value of 8 mg/L and a MIC_90_ value of >8 mg/L (Table 1); thus, preventing the definition of an ECOFF. Of note, few vancomycin- and teicoplanin-resistant *vanA*-type VRE demonstrated dalbavancin MICs in the lower range of ≤0.5 mg/L (*n* = 6) (Figure 1A, Supplementary Table S1). Growth deficiencies in MH broth and a low resistance to teicoplanin (8 or 16 mg/L) were observed for almost all of these isolates, as can also be found for some of the now prevalent VRE isolates in Germany (based on experience at the NRC) (Supplementary Table S1). Dalbavancin-resistant and *vanA*-positive isolates generally showed vancomycin and teicoplanin MICs of >16 mg/L (Supplementary Table S1). A single *vanA* isolate that did not reveal growth deficiencies in MH broth was tested vancomycin- and teicoplanin-susceptible (MICs ≤ 1 mg/L) (Supplementary Table S1). This isolate demonstrated dalbavancin MICs of 0.125 and 0.064 mg/L when determined using BMD and MIC test strips, respectively (Supplementary Table S1). Detailed sequence analysis of the corresponding *vanA* gene cluster revealed a 5′ truncated *vanX* gene (data not shown). For isolates that showed deficiencies in growth behavior (*n* = 5), assessment of their *vanA* clusters did not reveal any genetic changes that could potentially be associated with this unusual phenotype. Prolonged incubation for up to 48 h produced dalbavancin MICs in the resistant range for four out of five isolates (Supplementary Table S1).

### 2.3. Determination of Dalbavancin MICs by MIC Gradient Strip Testing

For reasons of comparison and the determination of an ECOFF, we converted the MICs of MIC gradient strips into doubling dilution as found in BMD. Furthermore, MIC values of 16, 32 and >32 mg/L were downsized to a maximum of >8 mg/L (Supplementary Table S1). In case of deviations between the BMD and MIC Test Strips, the exact MICs are shown in brackets. Vancomycin-susceptible and *van*-negative *E. faecium* isolates (*n* = 25) demonstrated a MIC_50_ value of 0.032 mg/L and a MIC_90_ of 0.064 mg/L (Table 1). The ECOFF was 0.25 mg/L (Table 1, Figure 1B). Vancomycin-resistant and *vanB*-positive *E. faecium* isolates (*n* = 101) demonstrated a MIC_50_ value of 0.016 mg/L and a MIC_90_ of 0.032 mg/L (Table 1), which was one dilution step below that of *van*-negative *E. faecium* isolates. The ECOFF was 0.125 mg/L (Table 1, Figure 1B). All *van*-negative and *vanB*-positive *E. faecium* isolates revealed a gradient strip MICs of ≤0.25 mg/L (Figure 1B, Supplementary Table S1). *E. faecium* possessing *vanA* (*n* = 50) showed a MIC_50_ value of >8 mg/L (>32 mg/L) and a MIC_90_ of >8 mg/L (>32 mg/L) (Table 1), thus preventing the definition of an ECOFF. In accordance with results from BMD, few vancomycin- and teicoplanin-resistant *vanA* isolates demonstrated dalbavancin gradient strip MICs in the lower range of ≤0.5 mg/L (*n* = 4) (Figure 1B, Supplementary Table S1). Using the macro method (McFarland 2.0, BHI agar, 48 h incubation), only two *vanA* isolates demonstrated dalbavancin MICs ≤0.5 mg/L (Supplementary Table S1), one of which carried the truncated *vanX* gene (see above). Isolates showing growth within a defined inhibition zone were categorized as dalbavancin-resistant with a MIC >32 mg/L (Supplementary Figure S2, see discussion).

### 2.4. Comparing Results of Broth Microdilution and MIC Test Strips

Dalbavancin MICs derived from BMD and MIC gradient strip tests (BMD adapted MIC values) were in good overall concordance (Supplementary Figure S3). For *van*-negative and *vanB*-positive isolates, however, the MIC_50_/ MIC_90_ values of MIC gradient strips were slightly lower when compared to the BMD results (Table 1). Thus, the ECOFF was one doubling dilution higher when determined using BMD (Table 1). Irrespective of the testing method, all *van*-negative and all *vanB*-positive *E. faecium* isolates demonstrated MICs of ≤0.25 mg/L (CLSI breakpoint for E. faecalis). Dalbavancin MIC values for *vanA*-positive isolates ranged from ≤0.008 to >8 mg/L in BMD and 0.016 mg/L (0.012 mg/L) to >8 mg/L (>32 mg/L) for the MIC test strips (Supplementary Figure S3C). In contrast to BMD, the majority of the *vanA*-positive *E. faecium* isolates exhibited dalbavancin MIC values >8 mg/L (>32 mg/L) when determined using MIC gradient test strips (Supplementary Figure S3C).

## 3. Discussion

To identify a dalbavancin ECOFF for *E. faecium*, we determined the dalbavancin MICs for a total of 176 well-characterized, vancomycin-sensitive, *vanA*- or *vanB*-positive *E. faecium* isolates.

To date, available data on the in vitro efficacy of dalbavancin mainly come from complementary clinical studies [22,23,24,25,26]. However, within these studies, enterococci were hardly considered, and many of these studies have been conducted in the USA, where the majority of *E. faecium* infections is caused by *vanA*-type VRE. Since dalbavancin has no in vitro activity against *vanA*-carrying *E. faecium*, VRE data were not included in most clinical or registration trials. As a result, data on *E. faecium* are generally scarce, and knowledge about the in vitro activity of dalbavancin against different *van* genotypes is almost non-existent.

The determination of dalbavancin MICs is challenging for several reasons. First, all lipoglycopeptides show a facile adsorption to plastic surfaces [27]. The strength of the surface adhesion depends on the plastic material and thus on the type of microtiter plates used in the experiment. In order to prevent the depletion of the active substance from the test medium, the administration of polysorbate 80 (Tween 80) is essential. Second, readout of dalbavancin MICs can be challenging for BMD and MIC gradient strip tests because some clinical VRE strains may demonstrate growth deficiencies in standard media. This effect, in conjunction with an inducible resistance mechanism like VanA, can lead to delayed or weak growth in susceptibility tests. In BMD, this growth behavior is manifested by either very weak or diffuse colony growth when compared to the antibiotic-free growth control. For MIC gradient strip assays, the lack of growth leads to effects reminiscent of “heteroresistance”, which is detected either by microcolony growth in an otherwise clear inhibition zone (Supplementary Figure S2B) or a visible inhibition zone, but overall shaded growth within this zone (Supplementary Figure S2A). Third, dalbavancin is not available for the commercial test panels of automated systems, requiring manual handling, sample preparation, and readout.

The majority of the *vanA*-positive *E. faecium* isolates showed dalbavancin MICs of >8 mg/L (BMD) (Figure 1A, Supplementary Table S1). In contrast to *vanB*-type resistance, the regulatory system of *vanA*-positive VRE are sensitive to induction by teicoplanin and dalbavancin, resulting in the synthesis of modified peptidoglycan precursors and thus a resistance against all (lipo-) glycopeptides [28]. A few *vanA*-type VRE also revealed dalbavancin MICs in the lower range (≤0.5 mg/L) (Figure 1A, Supplementary Table S1). Similarly, data from a study by Zhanel et al. indicated a wide range of dalbavancin MICs for *vanA*-type *E. faecium* isolates reaching from 0.03 mg/L to >32 mg/L [17]. However, the authors did not address this aspect and did not provide any explanation as to whether and why dalbavancin might be active against some *vanA*-type isolates. Our research showed that five out of six *vanA* isolates with a dalbavancin MIC of ≤0.5 mg/L revealed weakened growth in MH broth. As such, it could be argued that more general growth deficiencies were responsible for the comparably low dalbavancin MICs. In case of a weakened growth, prolonged incubation of up to 48 h facilitated BMD MIC determination and prevented the detection of false-negative results (Supplementary Table S1). Similarly, a supplementation with 10% rich media such as BHI in combination with an increased inoculum (McFarland 2.0) and 48 h incubation period improved experimental outcomes when using MIC test strips (Supplementary Table S1). Both variations could be considered as a deviation from susceptibility testing guidelines and standards. Interestingly, one *vanA* isolate showed dalbavancin MICs in the susceptible range regardless of the method used, but no growth defect was observed (Supplementary Table S1). Subsequent in silico analysis revealed a trunked *vanX* gene, most probably leading to a non-functional VanX protein. VanX is essential for glycopeptide resistance because it degrades the d-Ala-d-Ala dipeptide into its individual components and thus prevents the formation of glycopeptide susceptible cell wall precursors.

The majority of published studies that have evaluated the in vitro activity of dalbavancin have focused either only on *E. faecalis* or did not distinguish between *E. faecalis* and *E. faecium* and/or the different *van* genotypes [21,29,30]. Therefore, these studies do not allow for a comparison with our data. With the beginning of the 2010s, however, several US medical centers presented results on the in vitro activity of dalbavancin for some *E. faecium* isolates, including VRE. A study conducted by Sader et al. included 30 *E. faecium* isolates, 18 of which carried the *vanA* gene. Of these, all but one isolate demonstrated a dalbavancin MIC of >4 mg/L [21]. Similarly, Jones and colleagues published a study on the potency profiles for dalbavancin and found that all included *vanA*-type enterococci (19 *E. faecium*, 6 *E. faecalis*) had dalbavancin MICs of ≥4 mg/L [20].

Due to the general prevalence of *vanA*-type resistance in clinical *E. faecium* and *E. faecalis* isolates for many years, comprehensive data on dalbavancin resistance in *vanB*-type enterococci are generally scarce [17,20,21,30]. A recently performed and yet unpublished study determined the dalbavancin MICs of clinical VRE isolates (including *vanA*- and *vanB*-type *E. faecium*) (Kresken et al., unpublished). Similar to our study, the MIC_50_ and MIC_90_ values for *vanB* isolates were 0.06 mg/L and 0.125 mg/L, respectively. For the *vanA*-positive isolates, the MIC_50_ was 8 mg/L, and the MIC_90_ was >8 mg/L. All of the *vanB* strains possessed dalbavancin MICs of ≤0.25 mg/L, and all *vanA* isolates revealed dalbavancin MICs of ≥4 mg/L.

In conclusion, we determined dalbavancin MICs for the largest and best-described strain collection of clinical *E. faecium* isolates, comprising both vancomycin-susceptible and vancomycin-resistant isolates of the *vanA*- and *vanB*-types. Results are reliable and consistent across different methodologies when dalbavancin susceptibility testing is performed according to the recommended guidelines. Based on the here provided and recently published data, we suggest a tentative dalbavancin ECOFF of 0.25 mg/L or a breakpoint of S ≤ 0.25 mg/L for *E. faecium*. Using this ECOFF or breakpoint, *van*-negative and *vanB*-positive *E. faecium* isolates would be considered dalbavancin-susceptible, while most *vanA*-type VRE, with the exception of a few, would be categorized as dalbavancin-resistant.

## 4. Materials and Methods

*Strain collection.* Within the present study, we included 176 clinical *E. faecium* isolates collected between January 2018 and December 2019 (Supplementary Table S1). All of the isolates derived from cases of bacteremia/blood stream infections and were submitted from clinical diagnostic laboratories to the NRC.

*Susceptibility testing.* We assessed susceptibilities to vancomycin and teicoplanin using BMD. Testing was performed according to EUCAST guidelines. EUCAST v8 and v9 standards were applied to categorize the MIC data into susceptible (S) and resistant (R). The MIC of dalbavancin (Sigma-Aldrich, St. Louis, USA) was determined by BMD using cation-adjusted Mueller–Hinton broth (Becton-Dickinson, Heidelberg, Germany) as described recently [18]. Deviating from this procedure, microdilution panels contained serial 2-fold dilutions of dalbavancin ranging from 8 to 0.008 mg/L. In order to alleviate adherence of the dalbavancin to the plastic surfaces of microtiter plates (Greiner Bio-One, Frickenhausen, Germany), the solution contained a final concentration of 0.002% polysorbate 80 (Tween 80; Sigma-Aldrich/Merck, Taufkirchen, Germany). In addition to BMD testing, the dalbavancin MICs were determined through a MIC gradient strip test on MH agar (Becton-Dickinson) according to the manufacturer’s instructions (Liofilchem, Roseto degli Abruzzi, Italy (http://www.liofilchem.net/login.area.mic/technical_sheets/MTS38.pdf, accessed on 22 June 2021). For reasons of comparison, we also applied the macro method using a higher inoculum of McFarland 2.0, Brain Heart Infusion (BHI) agar and a prolonged incubation time of up to 48 h before readout. Since EUCAST does not provide a clinical breakpoint or an ECOFF for dalbavancin, we compared our MIC data with the CLSI breakpoint provided for *E. faecalis* (S ≤ 0.25 mg/L). Reference isolates were *S. aureus* ATCC29213 and *E. faecalis* ATCC29212.

*Determination of ECOFFs.* ECOFFs were determined through the use of ECOFFinder [31]. A threshold of 99% was chosen (MIC value that captures 99% of the modelled wild-type population) to increase the specificity for the wild-type population.

*Determination of van genotypes*. As part of the daily routine, the *van* genotypes were assessed by multiplex PCR as described recently [32] and verified through in vitro susceptibility testing to vancomycin and teicoplanin. Additionally, *van*-genotypes were derived from de novo assembled read-data (see next paragraph).

*NGS-based analysis*. At the NRC for Enterococci, all isolates from bacteremia and sepsis are routinely sequenced using NGS. Whole genome sequencing was conducted in paired-end mode using the NextSeq 550 workflow with a read length of 2 × 150 bp (Illumina, San Diego, CA, USA). The quality of the raw sequence data was checked using FastQC v0.11.5 [33]. Taxonomic read classification was verified by Kraken v0.10.6 [34]. For the purpose of genome reconstruction, Illumina reads were de novo assembled using SPAdes with default parameters [35]. BWA-MEM was used for assembly-remapping and -polishing [36]. MLST and cgMLST were performed with de novo assembled contigs and Ridom SeqSphere^+^ v6.0.0 (Ridom; Münster, Germany). De novo assembled contigs were screened for *vanA* or *vanB* using BLAST [37]. Corresponding contigs were annotated using a customized database of relevant sequences (*vanR***,** *vanS***,** *vanH***,** *vanA* and *vanX*) and aligned through the use of MAFFT and Geneious Prime v2020.2.3 [38].

## Figures and Tables

**Table 1:** Distribution of dalbavancin MICs for *E. faecium* isolates (*n* = 176). MIC gradient strip values were converted into doubling dilution as found in BMD, and values of 16, 32, and >32 mg/L were downsized to >8 mg/L (maximum measurable value by BMD).

Genotype/Method	*n*	MIC [mg/L]												MIC_50_ [mg/L]	MIC_90_ [mg/L]	ECOFF * [99%]	CLSI ** [% S]
		≤0.08	0.016	0.032	0.064	0.125	0.25	0.5	1	2	4	8	>8				
*vanA*, *BMD*	50	1		1		3		1			1	21	22	8	>8	-	10
*vanA*, *strip*	50			1	1	1	1	1					45	>8	>8	-	8
*vanB*, *BMD*	101	1	6	64	27	3								0.032	0.064	0.25	100
*vanB*, *strip*	101	6	45	44	6									0.016	0.032	0.125	100
*van*-, *BMD*	25		3	6	11	5								0.064	0.125	0.5	100
*van*-, *strip*	25		6	11	7		1							0.032	0.064	0.25	100

Legend: BMD, broth microdilution; strip, MIC gradient strip test. The vertical line indicates the CLSI breakpoint for *E. faecalis*. * ECOFFs were identified by the use ECOFFinder (EUCAST). ** We applied the susceptibility breakpoint for vancomycin-susceptible *E. faecalis* as provided CLSI.3.

## Data Availability

Raw reads datasets generated for this study can be found in the European Nucleotide Archive (https://www.ebi.ac.uk/ena; study accession number: PRJEB43771, accessed on 26 July 2021).

## Supplementary Materials

The following are available online at https://www.mdpi.com/article/10.3390/antibiotics10080915/s1, Figure S1: Distribution of clonal lineages according to MLST for *van*-negative, *vanB*- and *vanA*-positive *E. faecium* isolates, Figure S2: Exemplary illustration of dalbavancin MIC determination by the use of MIC Test Strips, Figure S3: Concordance of dalbavancin MICs obtained by BMD and MIC test strips, Table S1: Details on the strain collection.

## References

[1] Kramer T.S., Remschmidt C., Werner S., Behnke M., Schwab F., Werner G., Gastmeier P., Leistner R. (2018). The importance of adjusting for enterococcus species when assessing the burden of vancomycin resistance: A cohort study including over 1000 cases of enterococcal bloodstream infections. Antimicrob. Resist. Infect. Control.

[2] European Centre for Disease Prevention and Control (ECDC) (2019). Surveillance of Antimicrobial Resistance in Europe 2018.

[3] Ayobami O., Willrich N., Reuss A., Eckmanns T., Markwart R. (2020). The ongoing challenge of vancomycin-resistant Enterococcus faecium and Enterococcus faecalis in Europe: An epidemiological analysis of bloodstream infections. Emerg. Microbes Infect..

[4] Buetti N., Wassilew N., Rion V., Senn L., Gardiol C., Widmer A., Marschall J. (2019). Emergence of vancomycin-resistant enterococci in Switzerland: A nation-wide survey. Antimicrob. Resist. Infect. Control.

[5] Hammerum A.M., Justesen U.S., Pinholt M., Roer L., Kaya H., Worning P., Nygaard S., Kemp M., Clausen M.E., Nielsen K.L. (2019). Surveillance of vancomycin-resistant enterococci reveals shift in dominating clones and national spread of a vancomycin-variable vanA Enterococcus faecium ST1421-CT1134 clone, Denmark, 2015 to March 2019. Eurosurveillance.

[6] Hoffmann M., Yao K., Allard M., Sanchez M., Andersen L.P., Hasman H., Hammerum A.M. (2018). Complete Genome Sequence of a Vancomycin-Resistant Sequence Type 203 Enterococcus faecium Strain with vanA Belonging to Complex Type 859. Microbiol. Resour. Announc..

[7] Eisenberger D., Tuschak C., Werner M., Bogdan C., Bollinger T., Hossain H., Friedrich P., Hussein Z., Pohlmann C., Wurstl B. (2020). Whole-genome analysis of vancomycin-resistant *Enterococcus faecium* causing nosocomial outbreaks suggests the occurrence of few endemic clonal lineages in Bavaria, Germany. J. Antimicrob. Chemother..

[8] Falgenhauer L., Fritzenwanker M., Imirzalioglu C., Steul K., Scherer M., Rhine-Main V.s.g., Heudorf U., Chakraborty T. (2019). Near-ubiquitous presence of a vancomycin-resistant *Enterococcus faecium* ST117/CT71/vanB -clone in the Rhine-Main metropolitan area of Germany. Antimicrob Resist. Infect. Control..

[9] Sadowy E., Gawryszewska I., Kuch A., Żabicka D., Hryniewicz W. (2018). The changing epidemiology of VanB *Enterococcus faecium* in Poland. Eur. J. Clin. Microbiol. Infect. Dis..

[10] Mischnik A., Werner G., Bender J., Mutters N.T. (2019). Enterococci With Special Resistance Patterns—Epidemiology, Hygiene and Therapy. Deutsche Medizinische Wochenschrift.

[11] Bender J.K., Cattoir V., Hegstad K., Sadowy E., Coque T.M., Westh H., Hammerum A.M., Schaffer K., Burns K., Murchan S. (2018). Update on prevalence and mechanisms of resistance to linezolid, tigecycline and daptomycin in enterococci in Europe: Towards a common nomenclature. Drug Resist. Updates.

[12] Klare I., Bender J.K., Markwart R., Reuss A., Abu SIn M., Eckmanns T., Werner G. (2019). Properties, frequencies and distribution of vancomycin-resistant enterococci in Germany—Update 2017/2018. Epidemiol. Bull..

[13] Klare I., Fleige C., Geringer U., Thurmer A., Bender J., Mutters N.T., Mischnik A., Werner G. (2015). Increased frequency of linezolid resistance among clinical *Enterococcus faecium* isolates from German hospital patients. J. Glob. Antimicrob. Resist..

[14] Bender J.K., Klare I., Fleige C., Werner G. (2020). A Nosocomial Cluster of Tigecycline- and Vancomycin-Resistant *Enterococcus faecium* Isolates and the Impact of rpsJ and tet(M) Mutations on Tigecycline Resistance. Microb. Drug Resist..

[15] Fiedler S., Bender J.K., Klare I., Halbedel S., Grohmann E., Szewzyk U., Werner G. (2016). Tigecycline resistance in clinical isolates of *Enterococcus faecium* is mediated by an upregulation of plasmid-encoded tetracycline determinants tet(L) and tet(M). J. Antimicrob. Chemother..

[16] Holmes N.E., Ballard S.A., Lam M.M., Johnson P.D., Grayson M.L., Stinear T.P., Howden B.P. (2013). Genomic analysis of teicoplanin resistance emerging during treatment of vanB vancomycin-resistant Enterococcus faecium infections in solid organ transplant recipients including donor-derived cases. J. Antimicrob. Chemother..

[17] Zhanel G.G., Calic D., Schweizer F., Zelenitsky S., Adam H., Lagacé-Wiens P.R., Rubinstein E., Gin A.S., Hoban D.J., Karlowsky J.A. (2010). New lipoglycopeptides: A comparative review of dalbavancin, oritavancin and telavancin. Drugs.

[18] Arhin F.F., Belley A., McKay G.A., Moeck G. (2010). Characterization of the in vitro activity of novel lipoglycopeptide antibiotics. Curr. Protoc. Microbiol..

[19] Rosenthal S., Decano A.G., Bandali A., Lai D., Malat G.E., Bias T.E. (2018). Oritavancin (Orbactiv): A New-Generation Lipoglycopeptide for the Treatment Of Acute Bacterial Skin and Skin Structure Infections. Pharm. Ther..

[20] Jones R.N., Flamm R.K., Sader H.S. (2013). Surveillance of dalbavancin potency and spectrum in the United States (2012). Diagn. Microbiol. Infect. Dis..

[21] Sader H.S., Mendes R.E., Pfaller M.A., Flamm R.K. (2019). Antimicrobial activity of dalbavancin tested against Gram-positive organisms isolated from patients with infective endocarditis in US and European medical centres. J. Antimicrob. Chemother..

[22] Raad I., Darouiche R., Vazquez J., Lentnek A., Hachem R., Hanna H., Goldstein B., Henkel T., Seltzer E. (2005). Efficacy and safety of weekly dalbavancin therapy for catheter-related bloodstream infection caused by gram-positive pathogens. Clin. Infect. Dis..

[23] Morata L., Cobo J., Fernandez-Sampedro M., Vasco P.G., Ruano E., Lora-Tamayo J., Somolinos M.S., Ruano P.G., Nieto A.R., Arnaiz A. (2019). Safety and Efficacy of Prolonged Use of Dalbavancin in Bone and Joint Infections. Antimicrob. Agents Chemother..

[24] Wang Y., Wang J., Wang R., Li Y., Cai Y. (2020). Efficacy and safety of dalbavancin in the treatment of Gram-positive bacterial infections. J. Glob. Antimicrob. Resist..

[25] Rappo U., Puttagunta S., Shevchenko V., Shevchenko A., Jandourek A., Gonzalez P.L., Suen A., Casullo V.M., Melnick D., Miceli R. (2019). Dalbavancin for the Treatment of Osteomyelitis in Adult Patients: A Randomized Clinical Trial of Efficacy and Safety. Open Forum Infect. Dis..

[26] Biedenbach D.J., Bell J.M., Sader H.S., Turnidge J.D., Jones R.N. (2009). Activities of dalbavancin against a worldwide collection of 81,673 gram-positive bacterial isolates. Antimicrob. Agents Chemother..

[27] Kavanagh A., Ramu S., Gong Y., Cooper M.A., Blaskovich M.A.T. (2019). Effects of Microplate Type and Broth Additives on Microdilution MIC Susceptibility Assays. Antimicrob. Agents Chemother..

[28] Cetinkaya Y., Falk P., Mayhall C.G. (2000). Vancomycin-resistant enterococci. Clin. Microbiol. Rev..

[29] Pfaller M.A., Flamm R.K., Castanheira M., Sader H.S., Mendes R.E. (2018). Dalbavancin in-vitro activity obtained against Gram-positive clinical isolates causing bone and joint infections in US and European hospitals (2011–2016). Int. J. Antimicrob. Agents.

[30] Pfaller M.A., Mendes R.E., Sader H.S., Castanheira M., Flamm R.K. (2017). Activity of dalbavancin tested against Gram-positive clinical isolates causing skin and skin-structure infections in paediatric patients from US hospitals (2014–2015). J. Glob. Antimicrob. Resist..

[31] Turnidge J., Kahlmeter G., Kronvall G. (2006). Statistical characterisation of bacterial wild-type MIC value distributions and the determination of epidemiological cut-off values. Clin. Microbiol. Infect..

[32] Klare I., Konstabel C., Mueller-Bertling S., Werner G., Strommenger B., Kettlitz C., Borgmann S., Schulte B., Jonas D., Serr A. (2005). Spread of ampicillin/vancomycin-resistant *Enterococcus faecium* of the epidemic-virulent clonal complex-17 carrying the genes esp and hyl in German hospitals. Eur. J. Clin. Microbiol. Infect. Dis..

[33] Andrews S., Krueger S., Segonds-Pichon F., Biggins L., Krueger C., Wingett S. (2012). FastQC: A Quality Control Tool for High Throughput Sequence Data.

[34] Wood D.E., Salzberg S.L. (2014). Kraken: Ultrafast metagenomic sequence classification using exact alignments. Genome Biol..

[35] Bankevich A., Nurk S., Antipov D., Gurevich A.A., Dvorkin M., Kulikov A.S., Lesin V.M., Nikolenko S.I., Pham S., Prjibelski A.D. (2012). SPAdes: A new genome assembly algorithm and its applications to single-cell sequencing. J. Comput. Biol..

[36] Li H., Durbin R. (2009). Fast and accurate short read alignment with Burrows-Wheeler transform. Bioinformatics.

[37] Camacho C., Coulouris G., Avagyan V., Ma N., Papadopoulos J., Bealer K., Madden T.L. (2009). BLAST+: Architecture and applications. BMC Bioinform..

[38] Katoh K., Standley D.M. (2013). MAFFT multiple sequence alignment software version 7: Improvements in performance and usability. Mol. Biol. Evol..

