# Supplementary material for: Determination of a Tentative Epidemiological Cut-Off Value (ECOFF) for Dalbavancin and Enterococcus faecium
Source: Antibiotics (Basel). 2021 Jul 27;10(8):915. doi: 10.3390/antibiotics10080915 (PMC8388697; doi:10.3390/antibiotics10080915)
Supplement: Supplementary file 1 [file antibiotics-10-00915-s001.zip › Supplementary_Figure_S1.pdf]

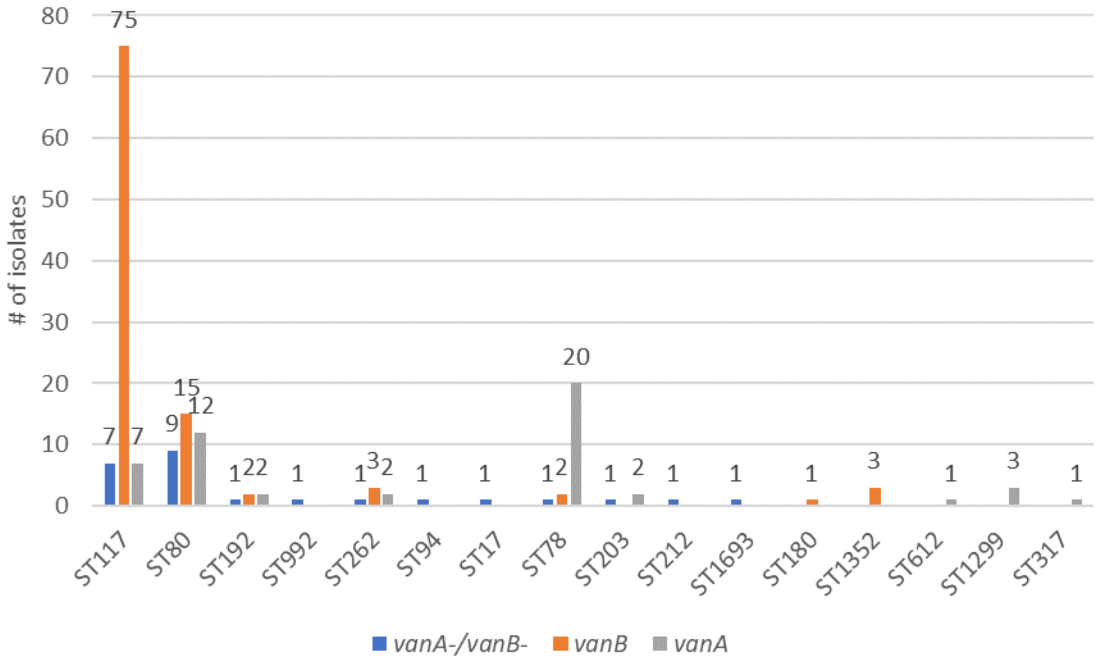

Supplementary Figure S1| Distribution of clonal lineages according to MLST for *van*-negative, *vanB*- and *vanA*-positive *E. faecium* isolates. The coloring of the bars corresponds to the given *van*-genotype (blue: *vanA*-/*vanB*-negative, orange: *vanB*, grey: *vanA*). The numbers above the bars indicate the number of isolates.
