# Supplementary material for: Determination of a Tentative Epidemiological Cut-Off Value (ECOFF) for Dalbavancin and Enterococcus faecium
Source: Antibiotics (Basel). 2021 Jul 27;10(8):915. doi: 10.3390/antibiotics10080915 (PMC8388697; doi:10.3390/antibiotics10080915)
Supplement: Supplementary file 1 [file antibiotics-10-00915-s001.zip › Supplementary_Figure_S2.pdf]

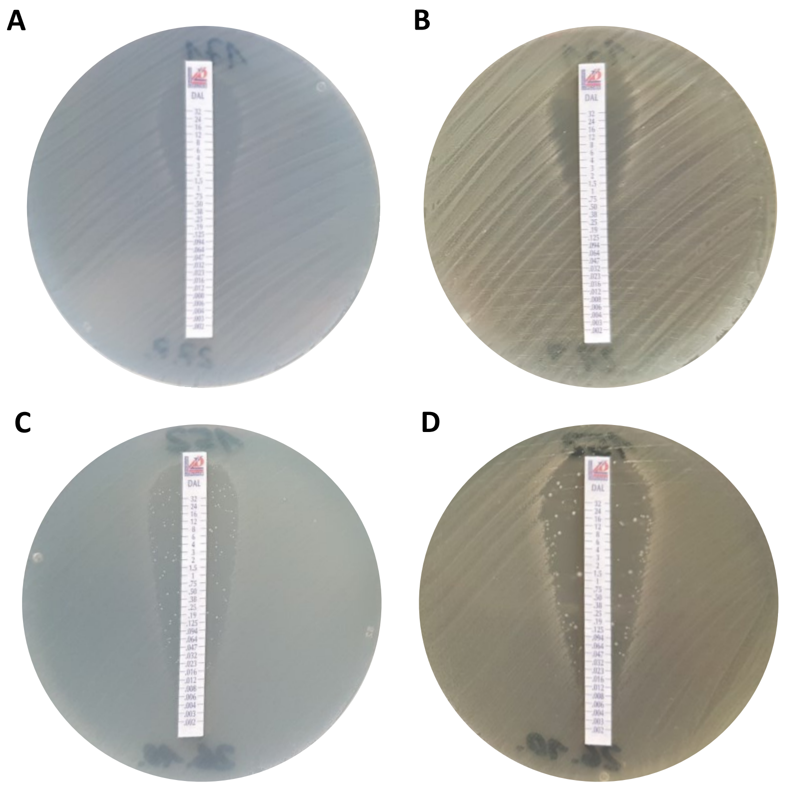

Supplementary Figure S2| Exemplary illustration of dalbavancin MIC determination by the use of MIC Test Strips. The test procedure was performed as recommended by the manufacturers instructions. *E. faecium* isolates UW17966 (A, B) and UW17873 (C, D) are shown after 48h incubation at 37°C using MH agar (A, C) or BHI agar (B, D). Due to shaded growth (A, B) and the formation of microcolonies (C, D) within an otherwise clear inhibition zone, isolates were classified as dalbavancin-resistant with MICs >32 mg/L.
