# Supplementary material for: Determination of a Tentative Epidemiological Cut-Off Value (ECOFF) for Dalbavancin and Enterococcus faecium
Source: Antibiotics (Basel). 2021 Jul 27;10(8):915. doi: 10.3390/antibiotics10080915 (PMC8388697; doi:10.3390/antibiotics10080915)
Supplement: Supplementary file 1 [file antibiotics-10-00915-s001.zip › Supplementary_Figure_S3.pdf]

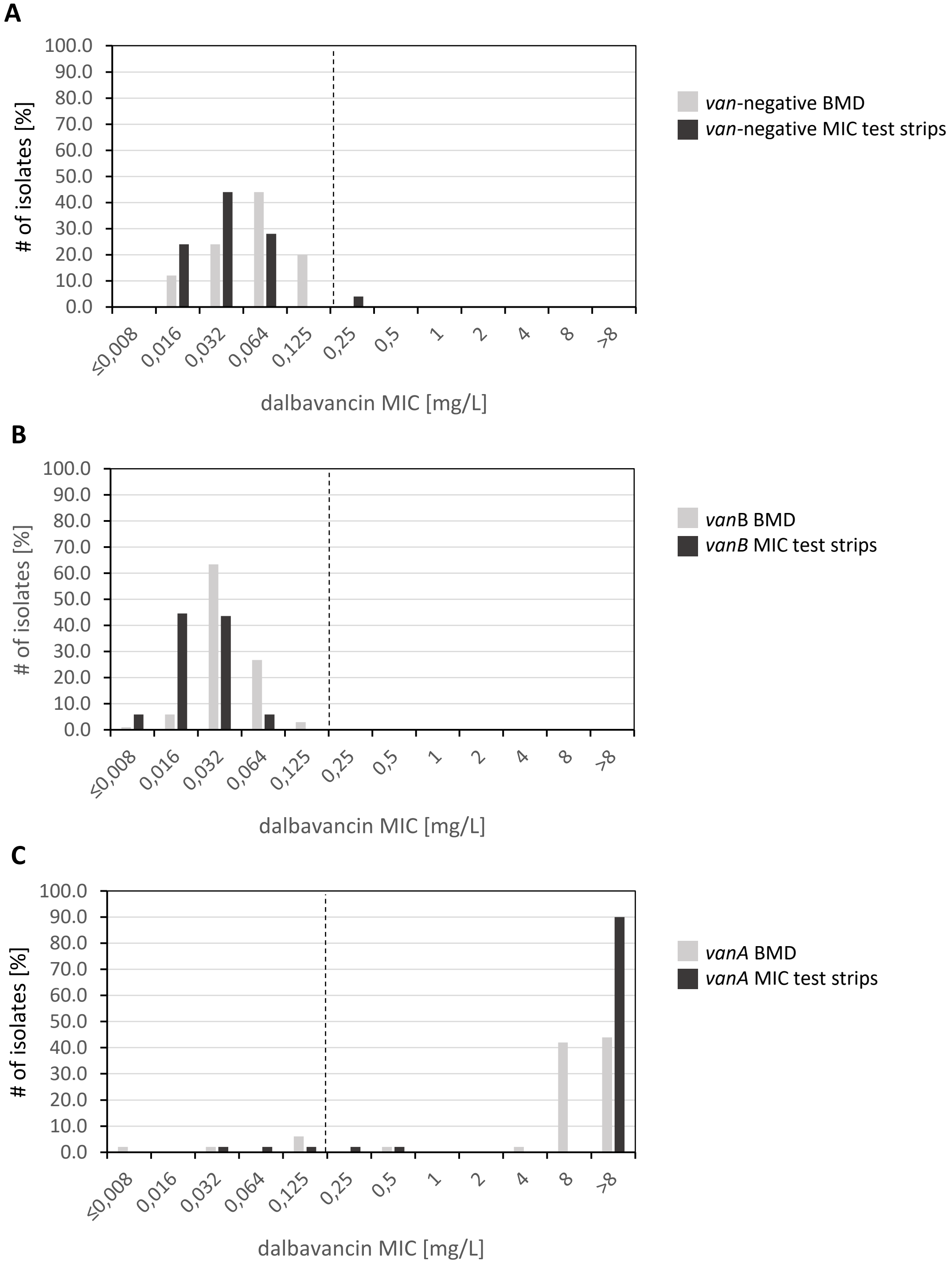

Supplementary Figure S3 | Concordance of dalbavancin MICs obtained by BMD and MIC test strips. MICs were obtained for 25 vancomycin-susceptible (A), 101 *vanB*-positive (B) and 50 *vanA*-positive (C) *E. faecium* isolates. The number of isolates with corresponding MICs is given in %. Gradient strip MICs were extrapolated to the next double dilution value equivalent to values of BMD and downsized to a maximum of >8mg/L. The MIC breakpoint for dalbavancin according to CLSI (*E. faecalis*) is indicated by the vertical dashed black line.
